# Virtual Reality for Addressing Depression and Anxiety: A Bibliometric Analysis

**DOI:** 10.3390/ijerph20095621

**Published:** 2023-04-24

**Authors:** Nuru Jingili, Solomon Sunday Oyelere, Frank Ojwang, Friday Joseph Agbo, Markus B. T. Nyström

**Affiliations:** 1 Department of Computer Science, Electrical and Space Engineering, Luleå University of Technology, 93187 Skellefteå, Sweden; 2 Faculty of Social Sciences, University of Lapland, 96300 Rovaniemi, Finland; 3 School of Computing, University of Eastern Finland, 80100 Joensuu, Finland; 4 School of Computing and Data Science, Willamette University, Salem, OR 97301, USA; 5 Department of Health, Education and Technology Division, Luleå University of Technology, 97187 Luleå, Sweden

**Keywords:** virtual reality, anxiety, depression, exposure therapy, cognitive behavioural therapy

## Abstract

Virtual reality is an emerging field in mental health and has gained widespread acceptance due to its potential to treat various disorders, such as anxiety and depression. This paper presents a bibliometric analysis of virtual reality (VR) use in addressing depression and anxiety from 1995 to 2022. The study analysed 1872 documents using the Scopus database, identifying the field’s most relevant journals and authors. The results indicate that using VR for addressing anxiety and depression is a multidisciplinary field with a wide variety of research topics, leading to significant collaborative research in this area. The *Annual Review of Cybertherapy and Telemedicine* was identified as the most relevant journal, while *Behavior Research and Therapy* was found to be the most cited journal. The analysis of keywords suggests that there is more research on using VR for anxiety and related disorders than for depression. Riva G. was identified as the top author in producing research outputs on VR-AD, and the University of Washington emerged as the leading institution in scientific outputs on VR-AD. Thematic and intellectual analyses helped identify the main themes within the research domain, providing valuable insight into the current and future directions of the field.

## 1. Introduction

The use of virtual reality technology for addressing anxiety and depression has been widely studied as a promising tool for preventing and treating anxiety and depression, as well as promoting mental well-being. In recent years, with the advancements in mobile and commercial VR capabilities, the implementation of virtual reality in anxiety and depression interventions has become more feasible and affordable. The emergence of the COVID-19 virus and the subsequent restrictions enacted by over 180 countries [1] have led to an increase in mental health conditions such as anxiety and depression [2,3,4,5,6], and many countries do not have adequate mental health resources and services [7]. As people were compelled to rely on technology to address restrictions, the pandemic has accelerated the use of technology-based interventions, including VR, for mental health treatment. The use of VR in addressing anxiety and depression has gained widespread acceptance due to its potential to provide remote tailored, immersive, and engaging experiences.

Several virtual reality (VR) interventions have recently been aimed at addressing mental health conditions and increasing the accessibility of mental health services to society [8,9,10,11,12,13]. VR can provide various opportunities to promote mental well-being, especially considering COVID-19 restrictions and the need for more accessible mental health services. VR is a technology that allows users to experience a completely different world by transporting them to a virtual world [8,10,14]. Through a combination of various technologies, such as a head-mounted display, headphones, and a joystick or hand-held controller, users can experience a fully immersive experience [8,13]. In VR, users can create responses that resemble real-world events as they interact with the world [10]. Due to VR’s potential to treat various mental health illnesses, VR has been widely used to treat a wide range of mental health conditions [8,13]. These mental health disorders include nervousness, anxiety, post-traumatic stress disorder, autistic spectrum disorders, paranoia, and phobia [8,9,10,11,12,13]. In addition, VR allows users to explore various coping exercises and techniques to help people with anxiety or depression overcome their problems by giving them a safe space to explore different scenarios without fear of consequences [9,15]. Despite the abundance of the literature on VR for anxiety and depression, the scholarly community has not taken advantage of the opportunity to assess its general knowledge objectively. However, a few studies have provided some insights into the status of VR used for anxiety and depression. For instance, Fodor et al. (2018) conducted a meta-analysis on VR interventions for anxiety and depression to explore the effects of VR interventions on treatment attrition. In another study, Carl et al. (2019) investigated the effectiveness of VR exposure therapy for anxiety-related disorders. Unfortunately, we could not find a single paper offering a bibliometric analysis of the former knowledge on virtual reality for addressing anxiety and depression (VR-AD). However, due to the topic’s significance, it is imperative to explore the bibliometric of the current literature to discover the gaps and trends. Therefore, this paper presents 1872 articles on VR-AD published between 1995 and 2022.

In this paper, we focus on the following questions:

1. What is the current publication trend of research on VR for anxiety and depression?
2. What are the frequently discussed themes in the research on VR for anxiety and depression?
3. What is the knowledge structure of the study on VR for anxiety and depression?
4. What is the direction for future research?

## 2. Methods

### 2.1. Research Design and Article Selection

This study seeks to examine research in the context of an interdisciplinary domain of computing and health that requires careful planning. The overarching objective is to examine how state-of-the-art technology can enhance the mental health and well-being of users. Therefore, appropriate data collection venues and search keywords that were aligned with the study’s objectives, research questions, and overall aim were identified through multiple brainstorming sessions among the authors. As a result, the Scopus database was chosen for data collection, as it indexes a vast number of published articles, including almost all publication venues that other databases, such as Web of Science, contain [16]. Scopus also covers almost all relevant conferences and journals for this study [17], and it provides high-quality data representation and structure that is suitable for quantitative bibliometric studies [18].

The search keywords for this study were obtained by scanning through the metadata of studies focusing on VR applications in anxiety and depression. To keep the study more focused and to further limit any bias that could impact the study outcomes, the authors met and agreed that the selected keywords are a good representation of the research objectives and can provide meaningful search results. The search keywords include “virtual reality”, “anxiety”, and “depression”. Table 1 showcases how the search strings were combined and executed using the Scopus webpage search engine. The table also shows the results obtained from the initial search and how several inclusion and exclusion criteria were applied to obtain the final data used for the analysis. The data collection was conducted on 17 January 2023, with a data range that was defined up to year 2022.

### 2.2. Inclusion and Exclusion Criteria

**Figure 1:**
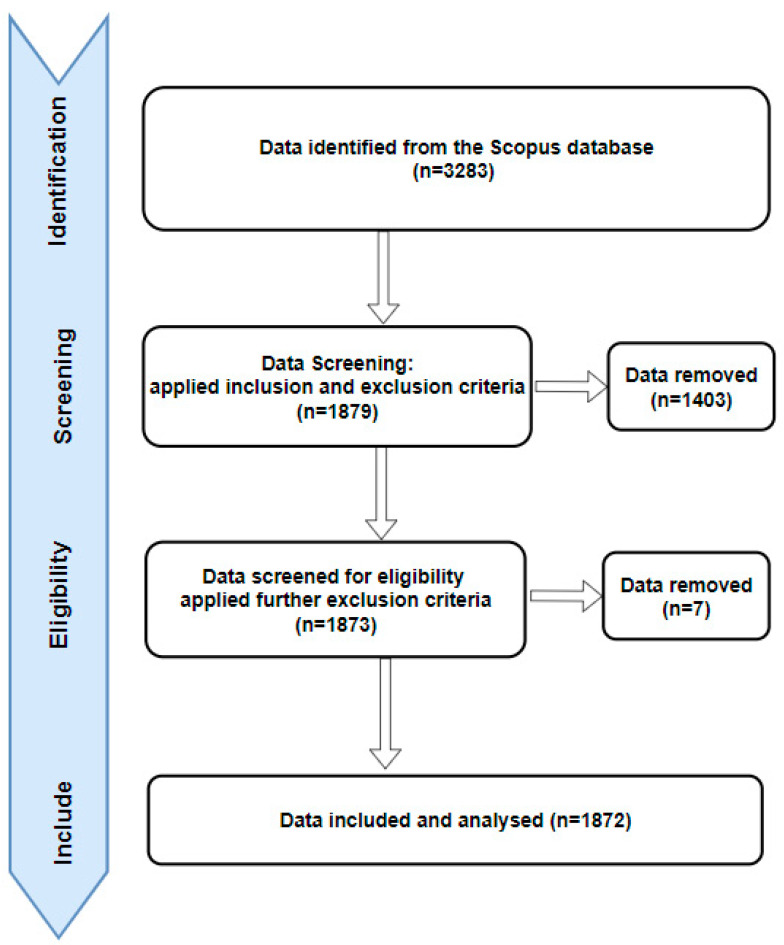
PRISMA flow diagram showing how data were collected and included in this study analysis.

The Prisma guidelines [19] are widely recognised procedures for conducting systematic reviews and meta-analyses to ensure a rigorous and transparent review process. In this study, the review was carried out according to these guidelines, as shown in Figure 1, to ensure that the research was conducted in a comprehensive and systematic manner.

To locate relevant studies for this review, the researchers used the Scopus database and searched for peer-reviewed journal articles using the keywords “Virtual reality” and “anxiety” or “depression”. The studies were included in the review if they met the following criteria: published in peer-reviewed journals, conferences, and book chapters, limited to articles with status “final”, written in English, and published any time up to 2022.

To ensure that the studies were of high quality, the researchers excluded studies that were not written in English, excluded documents classified as “editorial”, “Letters”, “Review”, “Conference Review”, “Short Survey”, and Erratum” and excluded articles in press. Using these criteria, the researchers aimed to ensure that the studies included in the review were of high quality and met the necessary standards for inclusion in a bibliometric review.

### 2.3. Data Extraction and Loading

The data included in this study were downloaded from the Scopus database in BibTeX file format. This file containing the dataset was processed with the R-library bibliometrix [20]. Specifically, the R-tool called biblioshiny developed by Aria and Cuccurullo [20] was employed to analyse the dataset. Biblioshiny is a web interface that allows for easy and flexible bibliometrix analysis with powerful visualisation that enables scholars to examine different metrics of a field.

### 2.4. Data Synthesis

Table 2 showcases the data synthesis by presenting the main information of the data analysed. Our search results revealed that the earliest paper on this topic was published in 1995. Therefore, in this study, we included data from the year 1995 up to 2022 for analysis. The synthesised dataset contains several sources such as journal, books, and conference proceedings.

## 3. Results and Discussion

### 3.1. Growth and Trends of VR-AD Research

**Figure 2:**
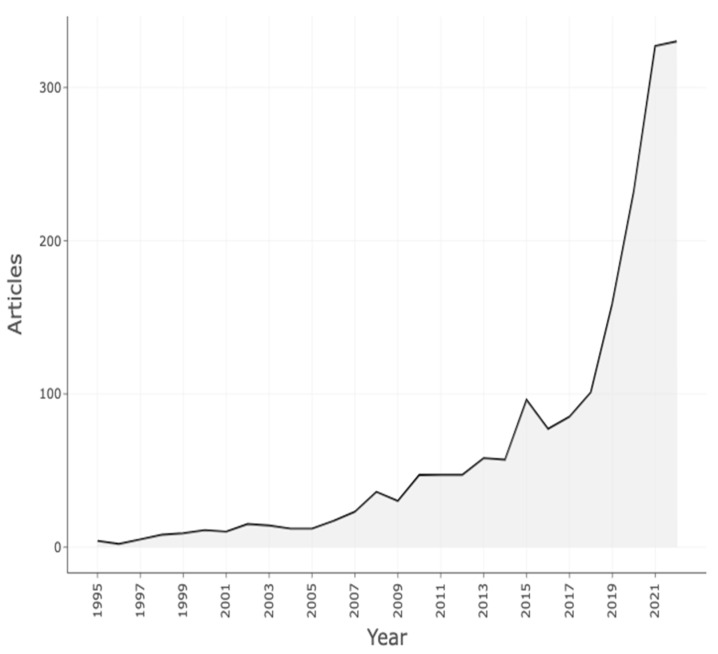
Annual scientific production of research on VR for anxiety and depression.

Figure 2 illustrates a growing trend and increased focus on VR-AD research. The COVID-19 pandemic in 2020 further exacerbated this situation, leading to an increased need for research that addresses the challenges associated with anxiety and depression during and after the pandemic. In 2021, there were 335 articles specifically focused on depression and anxiety, reflecting an apparent response to the impact of the COVID-19 pandemic. Research interest in using VR in addressing anxiety and depression has increased, with VR-AD research gaining particular attention since 2013. This can also be noted by the exponential VR-AD research between 2019 and 2021. This brings to the limelight the need to pay attention to the adoption of VR technology in preventing and managing depression and anxiety in the world today, especially among children and adolescents.

**Figure 3:**
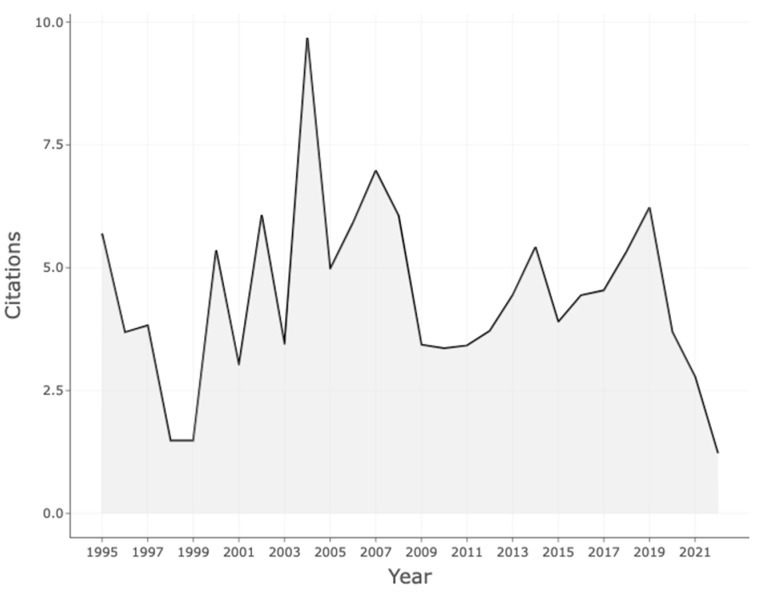
Average citation per year.

Figure 3 shows the trend in the citation of the articles with research focusing on VR-AD. The results showed that research interest in VR for anxiety and depression began in 1995 as citations started during this time. However, the citation of articles on VR-AD showed a fluctuating pattern over time, with some periods experiencing more research than others. Notably, in 2004, there was a significant increase in the number of citations on VR-AD publications.

### 3.2. Relevant Journals, Conferences, and Documents

**Figure 4:**
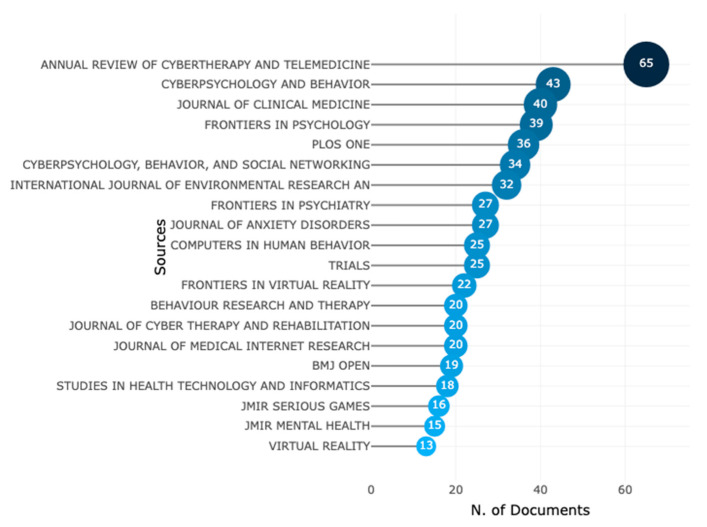
The 20 most relevant publication sources of VR for anxiety and depression.

**Figure 5:**
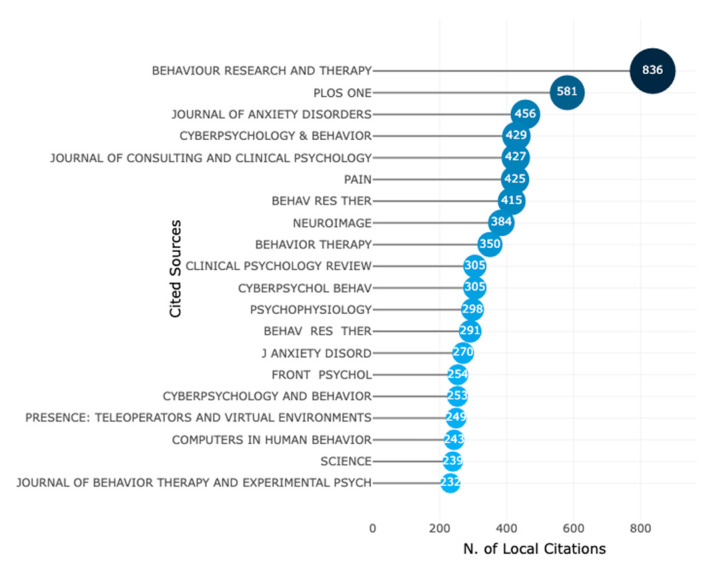
The 20 most local cited sources (from reference list).

In January 2023, data from Scopus was analysed to identify the leading sources of information on VR-AD research. The top 20 most relevant journals were identified and presented in Figure 4, with the *Annual Review of Cybertherapy and Telemedicine* being the premier source of information. Other notable sources included *Cyberpsychology and Behavior*, *Journal of Clinical Medicine*, *Frontiers in Psychology* and *PLOS ONE*. Additionally, the analysis revealed that the journal *Behaviour Research and Therapy* was the most frequently cited source, as shown in Figure 5. Other commonly cited sources included *PLOS ONE*, *Journal of Anxiety Disorders*, and journals in health and psychology.

### 3.3. Keyword Analysis

**Figure 6:**
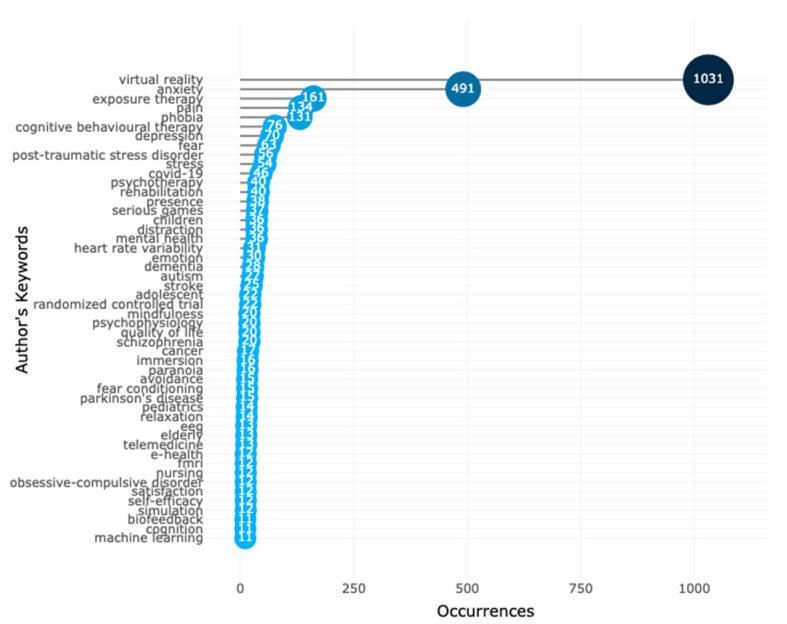
A visual representation of the commonly used keywords in VR-AD research. The use of these terms is among the highest in the field.

According to Song et al. [21], analysing the keywords authors use in their publications helps researchers identify the most relevant and interesting topics in the field. This is because the words used in these publications help them quickly identify the focus and trend of the issues in that area. Figure 6 shows the most frequently used words in research in VR-AD research in which anxiety appears more often (491 times) in the keywords than depression (70 times). This indicates that more research is being conducted on virtual reality and anxiety disorders than on depression. Other hot keywords in virtual reality for treating anxiety and depression research from 1995 to 2022 include virtual reality therapies such as virtual reality exposure therapy, psychotherapy, and cognitive behavioural therapy, research subjects such as children, methods of research studies such as randomised controlled trials, and related diagnoses such as pain, stress, social anxiety, mental health, and COVID-19. Overall, analysing the keywords used in VR-AD research can provide valuable insights into the field’s most relevant and popular topics. This information can help guide future research efforts and further our understanding of how VR technology can be used to address anxiety and depression.

**Figure 7:**
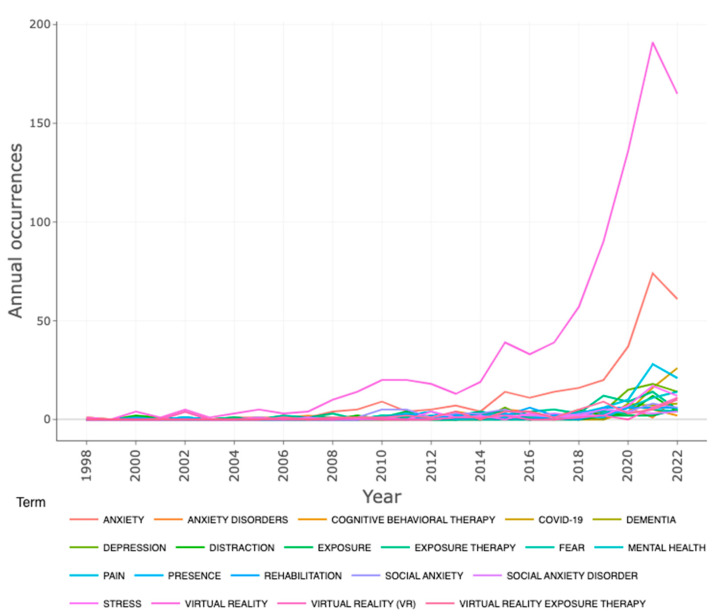
The dynamic view of the authors’ keywords over time.

Figure 7 shows the word dynamics of the most commonly used keywords by the authors in connection with VR-AD research. Most of these appeared in the literature from 1998 to 2008. However, in 2018, the multiple words used by them started to evolve and become more in line with the field’s overall trend. The most widely used keywords are related to anxiety disorders or anxiety. Furthermore, the usage of keywords such as COVID-19, dementia, cognitive behavioural therapy, depression, pain, and exposure therapy has also gained traction over the years. The sharp increase in usage of these keywords after 2020 suggests that these topics will continue to be a focus of VR-AD research.

**Figure 8:**
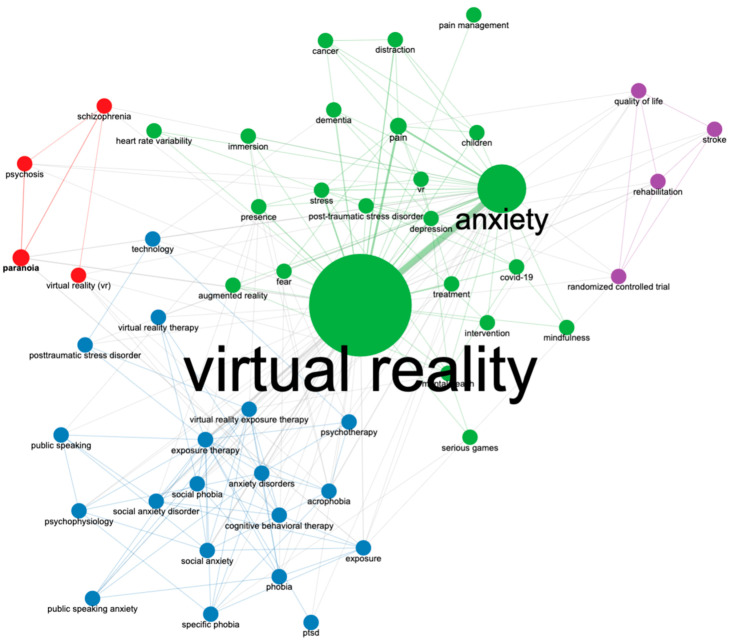
Co-occurrence network of keywords: The thick and thin lines represent the strong and weak associations between the various keywords.

This study also analysed the literature’s relationship between the various keywords and the co-occurrence networks in Figure 8. Keyword co-occurrence network analysis could help researchers better understand the field’s knowledge structure [22]. Figure 8 shows that the most substantial relationship is between virtual reality and anxiety, judging by the thickness of the line between them. This suggests that most research in this area involves anxiety and virtual reality. Other significant relationships are between virtual reality and anxiety with depression, pain, phobia, and exposure therapy.

### 3.4. Trending Topics and Thematic Analysis of VR-AD Research

**Figure 9:**
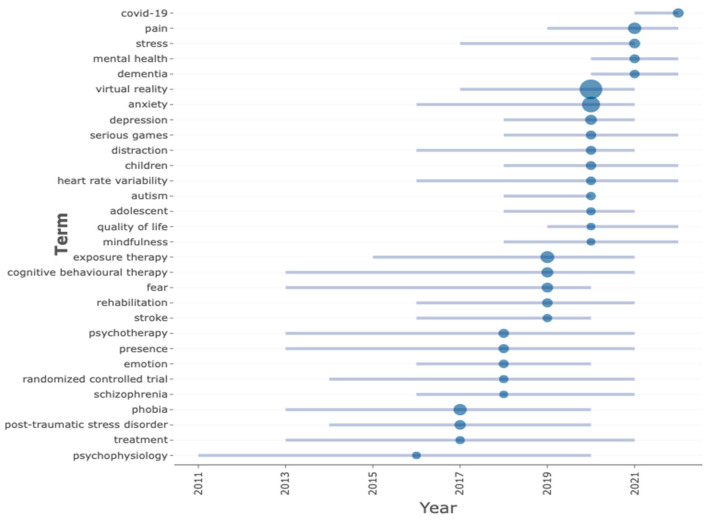
Trending topics of the last ten years.

In Figure 9, we evaluated trending topics based on the authors’ keywords by setting the data timespan to 1995–2022, minimum word frequency to 20, and 50 words per year. Keywords are connected to publication content and provide a basis for a field’s topical aspects [21]. This analysis gives us insight into VR-AD trends. From 2011 to 2020, “psychophysiology” was the most commonly used VR-AD keyword. From 2013 to 2020, “phobia”, “fear”, “treatment”, and “cognitive behavioural therapy” were widespread. “Randomized control studies” and “post-traumatic stress disorder” gained popularity in 2014 and remained relevant even with new topics emerging. From 2013 to 2020, “phobia”, “fear”, “treatment”, and “cognitive behavioural therapy” were widespread. “Randomized control studies” and “post-traumatic stress disorder” gained popularity in 2014 and remained relevant even with new topics emerging. “Exposure therapy” studies have increased since 2015. In 2016, VR-AD studies boomed concerning “anxiety”, “heart rate variability”, “schizophrenia”, “emotions”, “stroke”, “rehabilitation”, and “serious games”. VR-AD studies on “stress” increased in 2017. From 2018 to 2022, the number of VR-AD studies involving “children”, “adolescents”, “mindfulness”, “depression”, “serious games”, and “dementia” rose. The impact of COVID-19 on VR-AD investigations rose in 2021 and may continue to be a relevant topic.

**Figure 10:**
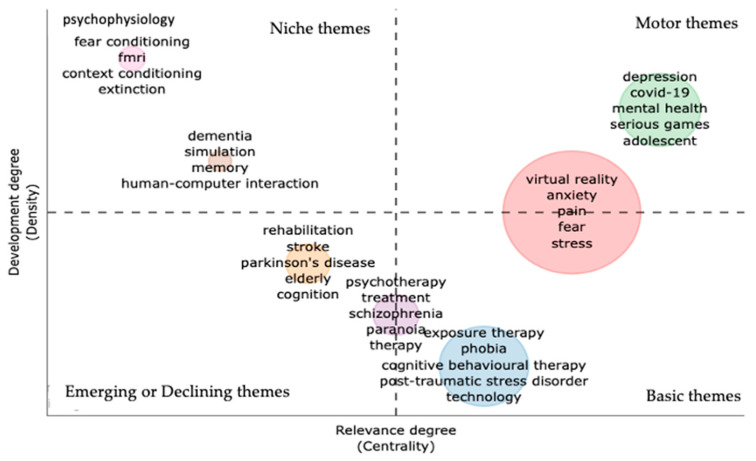
Thematic map with the top left quadrant representing the niche or specialised theme (Q1), the top right quadrant being the motor or driving theme (Q2), bottom left quadrant containing emerging or declining themes (Q3), and the bottom right quadrant consists of main or basic themes (Q4).

Figure 10 provides the thematic map of the VR-AD research. The goal of the thematic map is to understand the current state of the field and its future potential. This information is valuable for researchers and stakeholders in determining areas for future research development. The thematic analysis groups authors’ keywords and their connections to identify themes. These themes are evaluated based on two properties: density and centrality. Density is shown on the vertical axis and measures the cohesiveness among the nodes, while centrality, shown on the horizontal axis, measures the correlation among different topics [22]. For example, a higher number of connections with other nodes in the network results in higher centrality and importance, positioning the node at a crucial point in the network. Similarly, the density of a research field, represented by the cohesiveness of a node, indicates its ability to grow and maintain itself.

In generating the thematic map, we set the parameters as follows: number of words equal to 250, minimum cluster equal to 5, number of labels equal to 5, and the clustering algorithm used was Louvain. We uploaded a list of synonyms in the text editing option to avoid redundancy. The results show that there is an overlap on some topics between the basic or main themes in Q4 and the motor or driving themes in Q2. The keywords virtual reality, anxiety, pain, fear, and stress are both the main themes and driving themes. These are important themes in VR-AD research and are driving the research forward. Other driving themes found in the Q2 are depression, COVID-19, adolescents, serious games, and mental health. These driving themes have strong ties with the main themes and complement the research by adding a specialisation. Other main themes that are commonly discussed in VR-AD research are exposure therapy, different types of phobias, cognitive behavioural therapy, and post-traumatic stress disorder. There is also another overlap between the basic themes and emerging or disappearing themes, with the top keywords being psychotherapy, schizophrenia, and paranoia. On the emerging and disappearing themes, the found keywords are stroke, elderly, and Parkinson’s disease. This shows that there might be little research in VR-AD in regard to the keywords. On the specialised or niche themes of Q1, the important keywords are fMRI (functional magnetic resonance imaging), dementia, psychophysiology, human–computer interaction, fear and context conditioning, simulation, and memory. These keywords rarely appear in VR-AD research but have been noted to have a significant contribution to this research.

### 3.5. Publication by Countries

**Figure 11:**
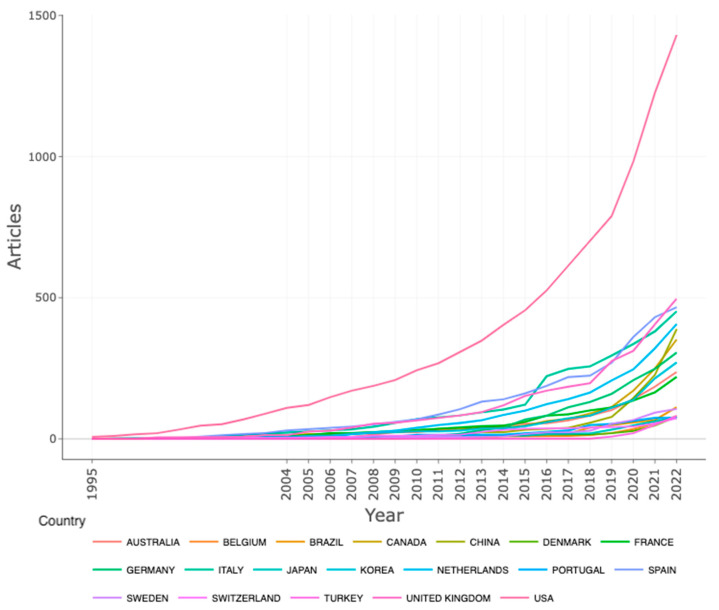
Countries’ production over time.

The study analysed the scientific output (publication quantity) of VR-AD research across different regions and countries. The results presented in Table 3 revealed that the United States of America (USA) has the most publications. The following top ten countries in scientific output on VR-AD research are the United Kingdom (UK), Spain, Italy, the Netherlands, China, Canada, Germany, Republic of Korea, Australia, France and Brazil. The majority of the countries involved in VR-AD research are European countries. Table 4, on the other hand, displays the top countries with the most cited publications, and once again, the USA is the most frequently cited nation. This can be partly explained by the high number of publications produced by the country. Figure 11 shows that for all topmost countries their research outputs have been steadily increasing over the years and have sharply increased in recent years. However, none of the African countries made it to the list of top 20 countries in terms of scientific output or most cited publications on VR-AD research. There could be several reasons why there is limited research on the use of virtual reality for mental health in Africa. For example, virtual reality technology is still developing in many African countries, and there may be limited resources, infrastructure and funding for researching and implementing mental health programs. Another reason is the stigma surrounding mental illness in some African countries. On the other hand, the USA, Canada, several European countries, China, and Australia are home to some of the leading technology companies and research institutions, which have the resources, lots of funding from governments and other sources and expertise to develop and study the use of virtual reality for mental health. Mental health is considered a priority issue in many developed societies, and there is a growing recognition of the importance of developing innovative treatments for mental health conditions. VR is seen as a potential tool to address mental health conditions by offering remote and self-management treatment options. Increased recognition, willingness to invest and prioritisation of mental health issues by governments, funding agencies and health organisations have also sparked the interest of many researchers in exploring innovative treatments for mental health conditions.

### 3.6. Authors

**Figure 12:**
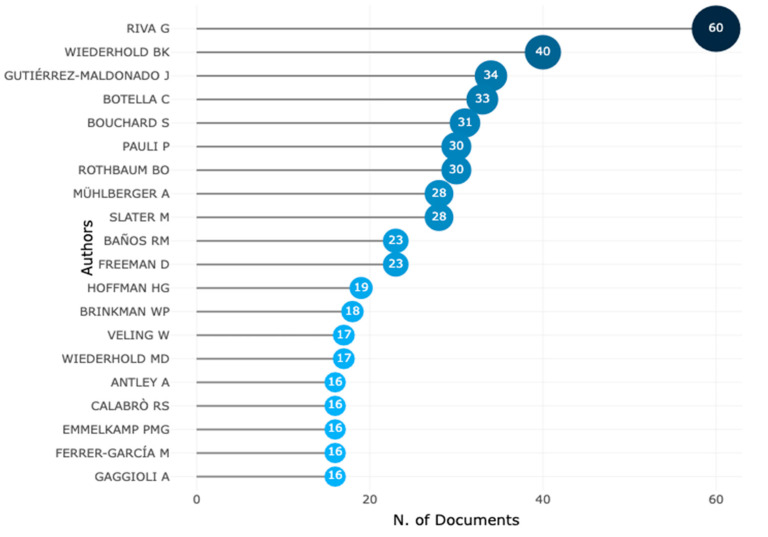
Most relevant authors in VR-AD research based on the number of documents.

**Figure 13:**
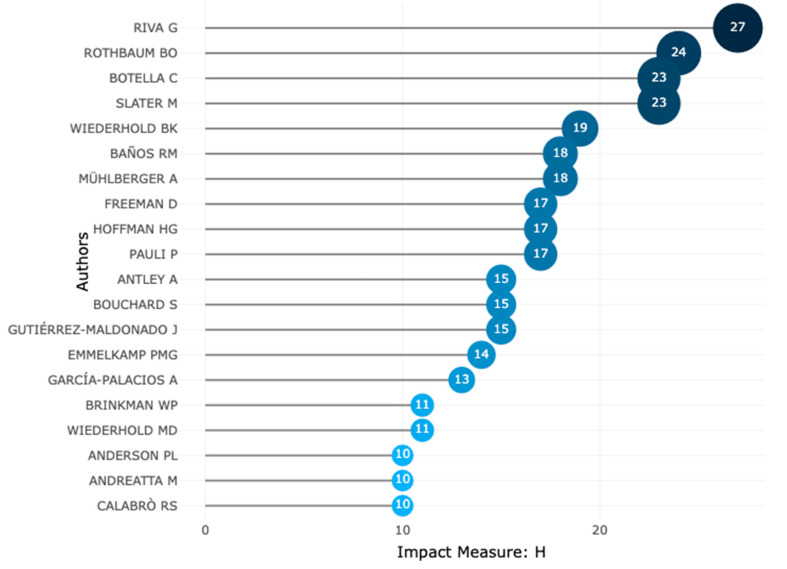
Authors’ local impact based on H-index.

**Figure 14:**
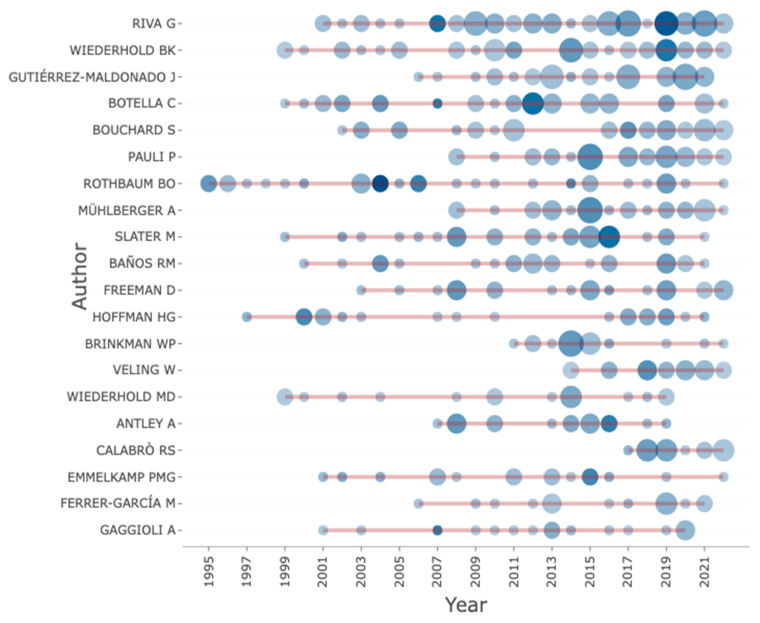
Authors’ production over time.

Figure 12 shows the top 20 most relevant authors in the VR-AD research domain from 1995 to 2022. The author who produced the most research documents in this field is Riva G., with a total of 60 documents. Following Riva G. is Wiederhold, with 40 papers focusing on VR research. It is worth noting that Riva G. also leads with the highest local impact based on an author H-index of 27, as shown in Figure 13, and she has had visibility since 2001.While some researchers such as Rothbaum and Wiederhold have been researching VR-AD since the 1990s, Riva G. has conducted more research on VR-AD, as shown in Figure 14. Botella C. and Wiederhold have also contributed significantly to VR-AD research with notable local impact based on their author H-index. Rothbaum, who has been in the research field with VR for the longest period since 1995, has produced 30 documents and has a local impact with an author H-index of 24. These findings suggest that certain authors have had a greater impact on the VR-AD research domain, particularly Riva G. and Wiederhold.

### 3.7. Institutions

**Figure 15:**
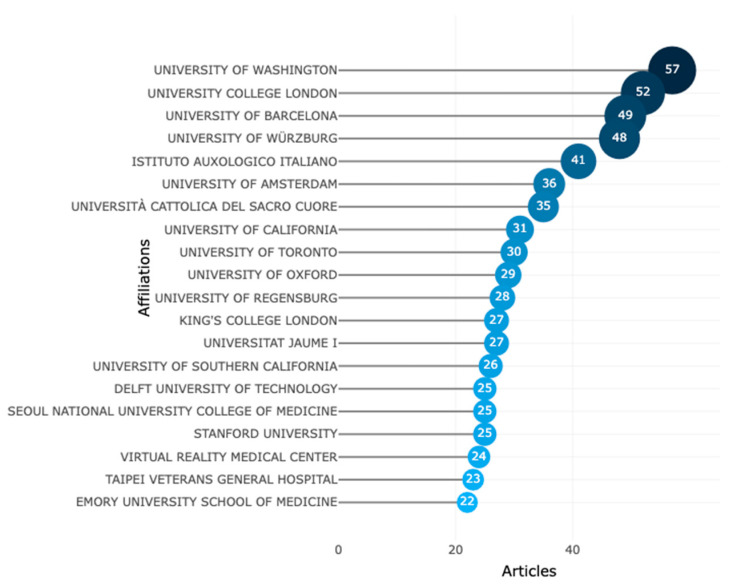
Most important institutions in VR-AD research.

The top 20 institutions that published the most research on VR-AD research are displayed in Figure 15. The University of Washington, located in the United States, leads with a total of 57 published articles exploring VR-AD. Other prominent institutions include the University College London in the United Kingdom, the University of Barcelona in Spain, the University of Wurzburg in Germany, and the Istituto Auxologico Italiano in Italy, with 52, 49, 48, and 41 published articles, respectively. This highlights the widespread adoption of VR technology in addressing anxiety and depression among developed countries in North America and Europe. In addition, other leading researchers in this field are based in universities throughout North America and Europe.

**Figure 16:**
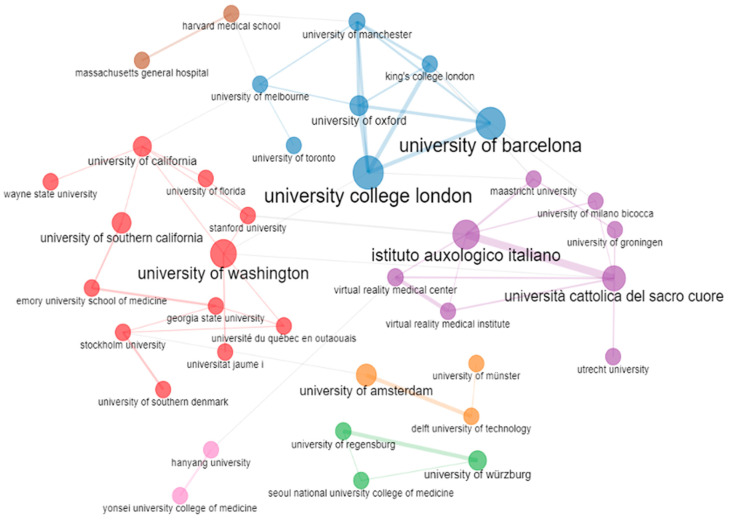
Institution collaboration network.

Figure 16 shows that the University of Washington in the USA collaborates the most with other universities in VR-AD research. The University of Washington collaborated with 11 universities, most of the partners being in the USA, followed by Europe. This explains the dominance of the University of Washington in VR-AD research. The University College London in the UK, the University of Barcelona in Spain, and the Istituto Auxologico Italiano in Italy have also collaborated with other research institutions. The University of Wurzburg has had relatively fewer partners compared to its peers in the top four research institutions that conduct research in the VR space.

### 3.8. Categorised Results of VR-AD Research

In this section, the quantitative analysis results are summarised and the current research status regarding the use of VR to treat anxiety and depression is presented. The findings obtained from the Scopus full-text database are classified into five categories, which are (i) research methods used in VR for treating anxiety and depression, (ii) types of VR used in VR-AD research, (iii) therapeutic methods used in VR-AD, (iv) research populations of VR-AD, and (v) diseases affected by VR-AD. Table 5 displays the frequency of words in a categorical format, which includes the actual names or their synonyms.

### 3.9. Research Methods of VR-AD

The analysis of the different research methods used in investigating the effectiveness of virtual reality (VR) treatments for anxiety and depression reveals that researchers employ a diverse range of methodologies to gain insights into the efficacy of these interventions. The most commonly used research method is the randomised controlled trial (RCT), which appeared 524 times in the authors’ keywords. RCTs are generally considered the most reliable method for assessing the efficacy of interventions [23]. RCTs provide a systematic and rigorous approach to assessing the effectiveness of VR-AD interventions. Their frequent use suggests that researchers place great importance on the reliability and validity of the data obtained through this methodology.

Other research methods used include pilot studies, case studies, double-blind studies, quantitative research methods, and observational methods. Pilot studies, which appeared 241 times, are useful for exploring new interventions and refining research methodologies. Case studies, which appeared 129 times, are also valuable as they enable researchers to understand the experiences of individuals who use VR treatments for anxiety and depression. Double-blind studies, which appeared 46 times, help minimise bias in research studies. In contrast, quantitative research methods, which appeared 18 times, enable researchers to measure the effectiveness of VR treatments for anxiety and depression using numerical data. Finally, observational techniques, which occurred 20 times, help gain insights into the experiences of individuals who use VR treatments for anxiety and depression in real-world settings.

The diverse range of research methods used in investigating the effectiveness of VR treatments for anxiety and depression highlights the complexity of this field and the need for a multidisciplinary research approach. While RCTs are the most commonly used research method, other methodologies, such as pilot studies, case studies, double-blind studies, quantitative research methods, and observational methods, also provide valuable insights into the effectiveness of these interventions. Furthermore, combining these methodologies can provide a comprehensive understanding of the potential benefits and limitations of VR-AD interventions.

### 3.10. Types of VR-AD

VR encompasses a broad range of technologies and applications, each with unique advantages and limitations. These VR types can be broadly divided into two categories: immersive VR and non-immersive VR. According to the study’s results, immersive virtual reality is the most frequently used type of VR in VR-AD research, with its keyword appearing 36 times. Immersive VR completely immerses users in a virtual environment, blocking out the view of the real world [24]. Typically, an HMD and other motion-tracking devices are used to create an immersive experience [8]. This study also found that the keyword “head-mounted display” appeared 15 times, emphasising the popularity of immersive VR experience.

Non-immersive VR, on the other hand, uses a computer screen, mobile phone, or projection to display the virtual environment rather than an HMD [25]. Although it is less immersive than immersive VR, non-immersive VR still provides a sense of presence in the virtual world. A 360-degree video is an example of non-immersive VR, with its keyword appearing 13 times in this study’s results. In 360-degree video, users can look around in any direction as if they were actually in the environment [26].

Augmented reality (AR) is closely associated with virtual reality, with its keyword appearing 26 times. AR is a technology that allows users to see and interact with the real environment [27]. By combining the virtual and real worlds, AR provides users with an enhanced sensory experience that allows them to see and interact with digital content more naturally and intuitively. AR can be classified as either AR or mixed reality (MR), both of which allow for the simultaneous display of virtual and real-world images, enabling users to interact with both simultaneously. Mixed reality is a type of technology that combines AR and VR elements to create a new type of experience. MR keywords appear 5 times in this study’s findings.

Each type of VR has its strengths and limitations. Immersive VR provides a highly immersive and interactive experience, but it requires expensive equipment. Non-immersive VR is more accessible and affordable, but it may not provide the same level of immersion as immersive VR.

### 3.11. Research Populations of VR-AD

The research population mainly involved in VR treatments for anxiety and depression varies depending on the specific study but generally includes individuals with a clinical diagnosis of anxiety or depression. These individuals may be recruited from clinical settings such as mental health clinics or hospitals or through online or community-based recruitment methods. In addition, some studies may focus on specific subgroups of individuals with anxiety and depression, such as those with social anxiety disorder, post-traumatic stress disorder (PTSD), or treatment-resistant depression. The age range of participants may also vary from children and adolescents to adults and older adults. It is important to note that research studies may have specific inclusion and exclusion criteria for determining participation eligibility.

The VR-AD research keywords were highest in the children-driven issues of anxiety and depression with 67 articles mentioning children, followed by 28 articles on adolescents, then 23 articles mentioning adults, 18 articles on the elderly, and, lastly, 8 articles mentioning youth. The children and adolescents find it easy to adapt to the technology, and thus, channelling potential solutions and interventions through the VR as a technology device is sensible for research involving children and adolescents. Children are also good beneficiaries of technological intervention as they are young and may not adhere to full disclosure when handling some issues with the adults but find it easy to self-express with the help of technology. An independent technology such as VR bridges the gap in an efficient and effective manner.

### 3.12. Therapeutic Methods in VR-AD

This study’s findings reveal that VR-AD research uses a diverse range of therapeutic methods for treating anxiety and depression. Exposure therapy, which involves gradually exposing patients to anxiety-provoking stimuli, is the most frequently used therapeutic method in VR-AD research, with 142 appearances. The high frequency of exposure therapy suggests that it is the most widely used therapeutic method for addressing anxiety and depression in VR-based interventions. Cognitive-behavioural therapy, which focuses on changing negative thought patterns and behaviours, is the second most commonly used therapeutic method in VR-AD research, with 77 appearances. This indicates that cognitive-behavioural therapy is also a widely used therapeutic method for addressing anxiety and depression in VR-based interventions.

Furthermore, the study reveals that other therapeutic methods, such as mindfulness-based therapy, biofeedback therapy, relaxation techniques, and eye movement desensitization and reprocessing (EMDR), are frequently used in VR-AD research. These findings suggest that various therapeutic methods can be used in VR-based interventions for anxiety and depression. In addition, the prominence of exposure therapy and cognitive-behavioural therapy in VR-AD research highlights their potential effectiveness in addressing anxiety and depression in this context.

### 3.13. Diseases Influenced by VR-AD

The diseases most influenced by VR are predominantly those that relate to anxiety; hence, there is a high frequency of terms such as fear and phobia, which sometimes are used to mean anxiety. According to the results, phobia, pain, fear, anxiety, depression, dementia, schizophrenia, and COVID-19 are some diseases and situations where VR-AD research is predominantly applied. Anxiety is the most researched topic, followed by phobia, pain, and fear. Depression, dementia, schizophrenia, and COVID-19 are other significant diseases utilising VR technology. The high research focus on the use of VR technology on anxiety-related disorders suggests that it may be more effective in treating anxiety-related disorders than depression.

The COVID-19 pandemic in 2020 has intensified the research focus on anxiety and depression-related disorders. The pandemic has had a profound impact on the mental health of individuals worldwide. Therefore, VR technology has become crucial to mitigate anxiety- and depression-related conditions.

## 4. Limitations and Benefits

This paper has several limitations that need to be noted. One of these is that researchers may use different keywords to describe the same concept, which could affect our search results. Another limitation is that we defined the synonyms manually and used Biblioshiny software to automatically set up the associated synonyms, which could also lead to errors in the data analysis and may cause minor variations in the results. Moreover, this review conducted bibliometric analysis on only one database, Scopus, and did not extend the search to other databases. Future studies on VR-AD studies could conduct searches in multiple databases.

This paper also has several strengths, one is that it gives an updated overview of the current state of research within the field. Another strength of this article is that it facilitates the possibility to identify areas that could benefit from more VR-AD research (e.g., other mental health disorders). A further strength of this study is that it can help researchers, who have interests in related areas, to identify and gain a clearer picture of competent researchers for future collaborations within this field.

## 5. Conclusions

The paper presents the results of a bibliometric analysis of VR-AD research. The authors believe that this is the first bibliometric analysis of research published on VR for anxiety and depression, highlighting the importance of further research in this area. The findings reveal that there is exponential growth in research outputs in this field, with more research on VR for anxiety and related disorders than for depression. Additionally, the findings show that the *Annual Review of Cybertherapy and Telemedicine* is the most relevant journal, while *Behavior Research and Therapy* is the most cited journal. Moreover, the results show that Riva G. and the University of Washington are identified as the top author and institution in producing research outputs on VR-AD.

Based on the findings of this study, there are several potential areas for future research in VR-AD. One possible direction is to explore the effectiveness of VR-based interventions for depression compared to anxiety and related disorders, as this study identified a larger amount of research on VR for anxiety than for depression. Another potential area of future research is to explore the underlying mechanisms of action for VR-based interventions. By examining how VR interventions affect cognitive, emotional, and behavioural processes in the context of anxiety and depression, researchers can better understand how these interventions work and which specific components of the interventions are most effective. Future studies may also focus on developing and evaluating specific VR interventions for different subtypes of anxiety and depression, focusing on personalisation and tailoring to individual needs. Another potential area for future research is the integration of VR with other treatment modalities, such as cognitive-behavioural therapy, to enhance the effectiveness of treatment for anxiety and depression. Finally, future studies may explore the potential for VR to address other mental health disorders beyond anxiety and depression, including phobias, post-traumatic stress disorder, and substance use disorders.

This study provides a valuable foundation for future investigations in the field of VR-AD. This study’s bibliometric analysis and thematic and intellectual analyses provide a structured overview of the research conducted in this field. The insights gained from this analysis can inform future research in this area. The current research on VR-AD suggests that this field will continue to grow and develop in the years to come, and the potential for VR to address other mental health disorders beyond anxiety and depression highlights the broad applicability of this technology in the field of mental health.

## Figures and Tables

**Table 1:** Data collection and selection criteria.

Search String	Result	Action (Inclusion)	Action (Exclusion)
TITLE-ABS-KEY (((“virtual reality” AND anxiety) OR (“virtual reality” AND depression))	3283 document results	Nothing	Nothing
TITLE-ABS-KEY ((((“virtual reality”) AND anxiety) OR ((“virtual reality”) AND depression))) AND (LIMIT-TO (PUBSTAGE, “final”)) AND (LIMIT-TO (DOCTYPE, “ar”)) AND (LIMIT-TO (LANGUAGE, “English”))	1879 document results	- Limited to articles in journal, conferences, and book chapters - limited to articles with status “final”. - Only documents written and published in English language	- excluded documents classified as “editorial”, “Letters”, “Review”, “Conference Review”, “Short Survey”, and Erratum”. - excluded “Article in Press”. - Documents written and published in other languages such as French, German, Spanish, Chinese, Portuguese, Russian, Hungarian, Italian, and Japanese
TITLE-ABS-KEY ((((“virtual reality”) AND anxiety) OR ((“virtual reality”) AND depression))) AND (LIMIT-TO (PUBSTAGE, “final”)) AND (LIMIT-TO (DOCTYPE, “ar”)) AND (LIMIT-TO (LANGUAGE, “English”)) AND (EXCLUDE (PUBYEAR, 2023))	1873 document results	- Nothing	- Excluded documents showing publication year as 2023 (1 outlier), book series, book, trade journal
TITLE-ABS-KEY ((((“virtual reality”) AND anxiety) OR ((“virtual reality”) AND depression))) AND (LIMIT-TO (PUBSTAGE, “final”))	1872	Final data included in the analysis	

**Table 2:** Summary of data included in the study analysis.

Description	Results
Main Information about Data	
Timespan	1995:2022
Sources (Journals, Books, etc)	785
Documents	1872
Annual Growth Rate %	17.76
Document Average Age	6.02
Average citations per doc	26.44
References	80,970
Document Contents	
Keywords Plus (ID)	7706
Author’s Keywords (DE)	3582
AUTHORS	
Authors	7619
Authors of single-authored docs	75
Authors Collaboration	
Single-authored docs	80
Co-Authors per Doc	5.7
International co-authorships %	23.45
Document Types	
article	1871

**Table 3:** Top 20 countries by publication.

Country	Frequency
USA	1431
UK	496
Spain	466
Italy	452
The Netherlands	407
China	389
Canada	352
Germany	306
Republic of Korea	271
Australia	237
France	220
Brazil	112
Sweden	106
Turkey	81
Belgium	80
Denmark	79
Japan	79
Portugal	74
Switzerland	73
Poland	64

**Table 4:** Top 20 most cited countries.

Country	Total Citations	Average Article Citations
USA	14,363	39.4
UK	3561	37.09
The Netherlands	3497	35.68
Italy	3321	35.71
Spain	3182	30.60
Germany	2326	23.03
Canada	1538	19.47
Australia	1531	26.86
Republic of Korea	1276	16.57
China	1229	11.49
France	1152	20.21
Switzerland	818	38.95
Fiji	691	345.50
Romania	602	46.31
Sweden	592	26.91
Denmark	548	30.44
Brazil	516	25.80
Israel	334	20.88
Portugal	327	19.24
Turkey	301	9.71

**Table 5:** Categorised results of VR-AD research.

Research Methods		Types of Virtual Reality Environments		Therapeutic Methods		Population		Diseases	
Randomised controlled trial	524	Immersive VR	36	Exposure therapy	142	Children	67	Dementia	32
Case study	129	Head Mounted Display/devise (HMD)	15	Cognitive behavioural therapy	77	Adolescents	28	Pain	140
Pilot study	241	360 Videos	13	Mindfulness	30	Youth	8	Anxiety	580
Questionnaire	396	Mixed reality	5	Biofeedback	14	Elderly	18	Phobia	167
Observation	20	Augmented reality	26	Relaxation	25	Adults	23	COVID-19	56
Double blind	46					Veterans	13	Fear	133
Quantitative study	18							Schizophrenia	24
								Depression	82
